# Clinical and microbiological characteristics of purulent and non-purulent cellulitis in hospitalized Taiwanese adults in the era of community-associated methicillin-resistant *Staphylococcus aureus*

**DOI:** 10.1186/s12879-015-1064-z

**Published:** 2015-08-05

**Authors:** Chun-Yuan Lee, Hung-Chin Tsai, Calvin M. Kunin, Susan Shin-Jung Lee, Yao-Shen Chen

**Affiliations:** Division of Infectious Diseases, Department of Medicine, Kaohsiung Veterans General Hospital, Kaohsiung, Taiwan; Faculty of Medicine, School of Medicine, National Yang-Ming University, Taipei, Taiwan; Department of Internal Medicine (CMK), Ohio State University, Columbus, Ohio and the University of Arizona, Tucson, Arizona USA; Graduate Institute of Science Education and Environmental Education, National Kaohsiung Normal University, Kaohsiung, Taiwan; University of Arizona, Tucson, AZ USA

**Keywords:** Cellulitis, Methicillin-resistant *Staphylococcus aureus*, Soft tissue infections, Staphylococcal skin infections

## Abstract

**Background:**

The risk factors, microbial etiology, differentiation, and clinical features of purulent and non-purulent cellulitis are not well defined in Taiwan.

**Methods:**

We conducted a retrospective cohort study of hospitalized adults with cellulitis in Taiwan in 2013. The demographic characteristics, underlying diseases, clinical manifestations, laboratory and microbiological findings, treatments, and outcomes were compared for patients with purulent and non-purulent cellulitis.

**Results:**

Of the 465 patients, 369 had non-purulent cellulitis and 96 had purulent cellulitis. The non-purulent group was significantly older (*p* = 0.001) and was more likely to have lower limb involvement (*p* < 0.001), tinea pedis (*p* = 0.003), stasis dermatitis (*p* = 0.025), a higher Charlson comorbidity score (*p* = 0.03), and recurrence at 6 months post-infection (*p* = 0.001) than the purulent group. The purulent group was more likely to have a wound (*p* < 0.001) and a longer hospital stay (*p* = 0.001) and duration of antimicrobial therapy (*p* = 0.003) than the non-purulent group. The etiological agent was identified in 35.5 % of the non-purulent cases, with β-hemolytic streptococci the most frequent cause (70.2 %). The etiological agent was identified in 83.3 % of the purulent cases, with *Staphylococcus aureus* the predominant pathogen (60 %): 50 % of these were methicillin-resistant *S. aureus* (MRSA). In multivariable analysis, purulent group (odds ratio (OR), 5.188; 95 % confidence interval (CI), 1.995–13.493; *p* = 0.001) was a positive predictor of MRSA. The prescribed antimicrobial agents were significantly different between the purulent and non-purulent groups, with penicillin the most frequently used antimicrobial agent in the non-purulent group (35.2 %), and oxacillin the most frequent in the purulent group (39.6 %). The appropriate antimicrobial agent was more frequently prescribed in the non-purulent group than in the purulent group (83.2 % vs. 53.8 %, *p* < 0.001).

**Conclusions:**

The epidemiology, clinical features, and microbiology of purulent and non-purulent cellulitis were significantly different in hospitalized Taiwanese adults. Purulence was a positive predictor of MRSA as the causal agent of cellulitis. These findings provide added support for the adoption of the IDSA guidelines for empirical antimicrobial therapy of cellulitis in Taiwan.

## Background

Cellulitis is a diffuse, spreading infection that involves the dermis and subcutaneous tissues of the skin. Predisposing factors include prior trauma (e.g. abrasion, laceration, puncture wounds), tinea pedis, stasis dermatitis, compromised lymphatic drainage, and venous insufficiency. It is often difficult to make a microbiological diagnosis because of the low recovery rate from needle aspirates, skin biopsies, and blood cultures [1, 2], and because of the identification of widely different causative organisms depending on the diagnostic tests used [3–5].

A confounding factor is the emergence of community-associated methicillin-resistant *Staphylococcus aureus* (CA-MRSA). Until the mid-1990s, MRSA infections were limited to hospitals. However, within the last decade, MRSA outbreaks have been seen in healthy individuals without connection to healthcare institutions [6]. CA-MRSA differs from healthcare-associated MRSA (HA-MRSA) in several ways, including a more limited antibiotic resistance profile, exotoxin gene profile (Panton Valentine leukocidin), type of staphylococcal cassette chromosome *mec* (SCC*mec*) gene, and clinical spectrum [7–9]. Infections of the skin and soft tissues account for 72–86 % of CA-MRSA cases [7, 8, 10]. The clinical manifestations range from mild skin and soft tissue infections, cutaneous abscesses, and purulent cellulitis [11, 12], to severe, life-threatening infections, including pyomyositis and myositis [13]. CA-MRSA is now the most important pathogen in purulent skin and soft tissue infections in the United States and Asia [11, 14].

The 2011 Infectious Diseases Society of America (IDSA) guidelines for the management of MRSA emphasize the need to distinguish purulent from non-purulent infections to clarify the relative contributions of β-hemolytic streptococci and *S. aureus* and their implications for antimicrobial therapy [15]. The epidemiology, microbiology, clinical manifestations, and contribution of CA-MRSA to non-purulent and purulent cellulitis are not well defined in Taiwan. In Taiwan, initial therapy for cellulitis is often empirical, using broad-spectrum coverage without adequate distinction between the microbiological etiology of non-purulent and purulent cellulitis.

This 1-year retrospective cohort study, conducted at Kaohsiung Veterans General Hospital in Southern Taiwan, was initiated to better understand the epidemiology, clinical features, and microbiology of non-purulent and purulent cellulitis in hospitalized Taiwanese adults. The findings are to be used as a basis for the development of guidelines for the diagnosis and management of cellulitis in Taiwan.

## Methods

### Study design

We conducted a 1-year retrospective cohort study, from January to December 2013, of the medical records of all adult patients admitted with the diagnosis of cellulitis to the Kaohsiung Veterans’ General Hospital (KVGH). KVGH is a 1200-bed general and tertiary care hospital located in Southern Taiwan. In our initial investigation, chart review of cases with International Classification of Disease, Ninth Revision, Clinical Modification (ICD-9-CM) codes of 729.4 (fasciitis), 728.86 (necrotizing fasciitis), and 608.83 (Fournier’s gangrene) were also carried out. Although some cases were initially diagnosed as cellulitis because of spreading erythema and swelling, these were finally diagnosed as deeper skin and soft tissue infections, and were not included in our study. The inclusion criteria were based on ICD-9-CM codes 528.3, 608.4, 681.00–681.9, and 682.0–682.9. Patients aged ≤20 years or those found not to have cellulitis on chart review were excluded from the study. The cases that met the inclusion criteria were further divided into non-purulent and purulent groups according to the definitions provided below. The two groups were then compared for demographic characteristics, underlying diseases, clinical manifestations, laboratory findings, diagnostic methods, pathogen, and outcomes. The study was approved by the ethical committee of Kaohsiung Veterans General Hospital (VGHKS14-CT12-01).

### Data collection

Hospital records were retrieved and relevant epidemiological, clinical, and microbiological data were compiled. Outcomes included length of hospital stay, antimicrobial therapy, 30-day all-cause and cellulitis-related mortality, and recurrent episodes of cellulitis at the initial site at 6 months post-infection.

### Definitions

Wound is defined as a pre-existing skin lesion at the time of skin infection onset, such as a chronic ulcer, or a trauma-related skin lesion, such as lacerations, abrasions, or punctures. Surgical site infection is not included in the present study.

Purulent cellulitis was initially defined as skin lesions associated with purulent drainage or exudate in the absence of a drainable abscess [15]. A cutaneous abscess was defined as a collection of pus within the dermis and deeper skin tissues [16]. Because of the difficulties associated with differentiating purulent cellulitis from cutaneous abscess in a retrospective chart review, we combined these two presentations into the purulent group. Non-purulent cellulitis was defined as cellulitis without purulent drainage or exudate or associated abscess [15].

CA-MRSA was defined as MRSA isolated from a patient who had none of the following established risk factors for HA-MRSA. These included isolation of MRSA > 48 h after hospital admission, history of prior hospitalization, surgery, dialysis, or residence in a long-term care facility within the previous year, the presence of an indwelling urinary catheter or a percutaneous device at the time of culture, or previous isolation of MRSA [17, 18]. Modified case definition, which excludes the previous isolation of MRSA as a criterion for HA-MR, was also analyzed in the present study [19].

An appropriate antimicrobial agent was defined as an antimicrobial agent to which the identified pathogen was susceptible.

Recurrence of cellulitis was defined as an attack of cellulitis in the same anatomical site within 6 months of cure of the previous episode of cellulitis.

### Microbiological studies

Bacteria were identified and antimicrobial susceptibility profiles were determined using a Vitek two automated system (bioMérieux, Canada) at the KVGH Clinical Microbiology Laboratory. All *S. aureus* isolates susceptible to oxacillin underwent confirmatory disk diffusion testing for cefoxitin susceptibility. MRSA isolates were tested for susceptibility to erythromycin, clindamycin, levofloxacin, rifampin, trimethoprim-sulfamethoxazole, and vancomycin. The D-test was performed to detect inducible clindamycin resistance for isolates that were resistant to erythromycin, but susceptible to clindamycin. All tests were performed and interpreted in accordance with Clinical and Laboratory Standards Institute guidelines (M100-S19).

Blood and pus cultures, skin biopsy, and serological antistreptolysin O titer (ASOT) reports were collected and analyzed. Coagulase-negative staphylococci, diphtheroids, and *Bacillus* species were considered to be blood culture contaminants when isolated from one culture bottle of a set, or when another set of blood culture bottles was sterile. A positive wound culture was defined as isolation of a bacterium from both a superficial and a deep site. The term β-hemolytic streptococci was used rather than *Streptococcus pyogenes* because the isolates were not differentiated by group. When both acute and convalescent ASOT results were not available to demonstrate a ≥2-fold increase in titer, a single positive ASOT with a titer of ≥200 U/mL, according to the manufacturer, was considered to be positive.

### Statistical analysis

All statistical analyses were performed using SPSS version 12.0 (SPSS, Chicago, IL, USA). Descriptive statistics were used to summarize the characteristics of the patients and the relative contributions of the non-purulent and purulent groups. Categorical variables were compared between these two groups using the *χ*^2^ or Fisher’s exact tests, and continuous variables were compared using an independent *t*-test. All tests were 2-tailed, and *p* < 0.05 was considered significant. Multivariable analyses of predictive factors for MRSA were performed in patients with a final pathogen diagnosis using the LOGISTIC procedure, and the candidate variables were identified as those having a univariate significance of *p* ≤ 0.10, those identified in a previous study, or those believed to be clinically meaningful.

## Results

### Demographic characteristics and underlying diseases in patients with cellulitis

We reviewed the medical records of 968 patients initially identified as having cellulitis. We excluded 503 cases (52.0 %) for the following reasons: infections other than cellulitis (382; 75.9 %), age ≤20 years (85; 16.9 %), non-infectious diseases (33; 6.6 %), and incomplete records (3; 0.6 %). The study population consisted of the remaining 465 patients, of which 369 (79.4 %) had non-purulent and 96 (20.6 %) had purulent cellulitis. Their demographic characteristics and underlying diseases are summarized in Table 1. The median age was 62 years (range, 20–96 years; SD ± 20.2); 61.3 % were men. The non-purulent group was significantly older (64 vs. 56 years, *p* = 0.001) and more likely to have tinea pedis (18.7 % vs. 6.3 %, *p* = 0.003), stasis dermatitis (7.0 % vs. 1.0 %, *p* = 0.025), and a higher Charlson comorbidity score (1.5 vs. 1.1, *p* = 0.03) than the purulent group. The purulent group was more likely to have a wound (44.9 % vs. 22.8 %, *p* < 0.001) than the non-purulent group. There were no significant differences between the two groups in gender composition.

**Table 1:** Demographic characteristics and underlying diseases among 465 cellulitis cases

	All (*n* = 465)	Non-purulent group (*n* = 369)	Purulent group (*n* = 96)	*P*-value
Age (years), mean (SD)	62 (20.2)	64 (19.4)	56 (21.9)	0.001
Male sex, no. (%)	285 (61.3)	221 (59.9)	64 (66.7)	0.225
Wound (%)	123 (26.5)	84 (22.8)	39 (40.9)	<0.001
Charlson comorbidity score, mean (SD)	1.4 (1.6)	1.5 (1.6)	1.1 (1.5)	0.03
Diabetes mellitus (%)	119 (25.9)	101 (27.4)	18 (18.8)	0.085
Autoimmune disease (%)	16 (3.4)	13 (3.5)	3 (3.1)	1.00
Solid malignancy (%)	65 (14)	56 (15.2)	9 (9.4)	0.144
Hematological malignancy (%)	13 (2.8)	12 (3.3)	1 (1.0)	0.484
Liver cirrhosis (%)	29 (6.2)	20 (5.4)	9 (9.4)	0.153
HIV infection (%)	4 (0.9)	3 (0.8)	1 (1.0)	1.00
Organ transplantation (%)	2 (0.4)	2 (0.5)	0 (0.0)	1.00
Chronic obstructive pulmonary disease (%)	13 (2.8)	9 (2.4)	4 (4.2)	0.318
Peripheral artery occlusive disease (%)	18 (3.9)	14 (3.8)	4 (4.2)	0.773
Coronary artery bypass graft (%)	11 (2.4)	9 (2.4)	2 (2.1)	1.00
Fracture (%)	9 (1.9)	9 (2.4)	0 (0.0)	0.215
Flap (%)	9 (1.9)	9 (2.4)	0 (0.0)	0.215
Lymph node dissection (%)	21 (4.5)	19 (5.1)	2 (2.1)	0.273
Tinea pedis (%)	75 (18.1)	69 (18.7)	6 (6.3)	0.003
Stasis dermatitis (%)	27 (5.8)	26 (7.0)	1 (1.0)	0.025

Data are no. (%) of patients, unless otherwise indicated

### Clinical and laboratory manifestations of patients with cellulitis

The clinical and laboratory findings for the 465 cases of cellulitis are summarized in Table 2. There were no significant differences between the two groups in frequency of sepsis at admission or clinical laboratory findings. Cellulitis was localized to the lower extremities, upper extremities, and head and neck in 78.7, 8.6, and 7.5 % of cases, respectively. Although lower extremity cellulitis was the most common site of infection in both groups, it was significantly more frequent in the non-purulent than the purulent group (82.4 % vs. 62.5 %, *p* < 0.001).

**Table 2:** Clinical manifestations, laboratory findings, and outcomes among 465 cellulitis cases

	All (*n* = 465)	Non-purulent group (*n* = 369)	Purulent group (*n* = 96)	*P*-value
Lower extremities involved (%)	364 (78.3)	304 (82.4)	60 (62.5)	<0.001
Sepsis at admission (%)	127 (27.3)	103 (27.9)	24 (25.0)	0.568
WBC, mean 10^9^/L (SD)	10.3 (4.9)	10.2 (5.0)	10.5 (4.8)	0.572
Hb (g/dL), mean (SD)	12.9 (2.2)	12.9 (2.2)	13.0 (2.3)	0.658
PLT/mm^3^ (SD)	200.6 (78.9)	195.0 (77.2)	221.9 (82)	0.003
Lactic acid (mmol/L), mean (SD)	1.8 (1.2)	1.8 (1.2)	1.9 (1.3)	0.917
Cr (mg/dL), mean (SD)	1.3 (1.7)	1.4 (1.9)	1.1 (0.7)	0.175
CK (U/L), mean (SD)	142.1 (343)	144.2 (368)	131.8 (165.9)	0.813
CRP (mg/dL), mean (SD)	6.3 (7.6)	6.3 (7.7)	6.3 (7.4)	0.993
GOT (IU/L), mean (range)	31.0 (23.6)	30.4 (19.8)	33.2 (34.4)	0.5
Albumin (g/dL), mean (SD)	3.1 (0.7)	3.1 (0.7)	3.2 (0.6)	0.71
Recurrence within 6 months (%)	57 (12.3)	54 (14.6)	3 (3.1)	0.001
Death within 30 days (%)	3 (0.6)	3 (0.8)	0 (0)	1.0
Length of stay, days (SD)	9 (6.5)	8 (5.8)	11 (8.1)	0.001
Duration of antimicrobial therapy, days (SD)	14 (9.7)	13 (9)	17 (12)	0.003

Data are number (%) of patients, unless otherwise indicated

*CK* creatine kinase, *Cr* creatinine, *CRP* C-reactive protein, *GOT* glutamate oxaloacetate transaminase, *Hb* hemoglobin, *PLT* platelet count, *SD* standard deviation

### Microbiological and serological studies

Microbiological and/or serological studies were performed in 91.6 % of cases in the non-purulent group and in 97.9 % of cases in the purulent group (Table 3). Wound culture, blood culture, ASOT, and skin biopsy were performed in 33.3, 75.5, 45.2, and 0.2 % of cases, respectively. One or more bacterial species were isolated from 110 of the 155 (71.0 %) wound cultures. Of the 110 patients with a positive wound culture, 15 (13.6 %) had superficial and 95 (86.4 %) had deep wounds. The prevalence of positive wound culture was higher in the purulent group than in the non-purulent group (88.6 % vs. 47.8 %, *p* < 0.001). Thirty-three of the 351 blood cultures (9.4 %) met the criterion for a positive culture. There was no significant difference in the prevalence of positive blood cultures between the non-purulent and purulent groups (8.2 % vs. 15.0 %, *p* = 0.103). Of the 210 patients with measured ASOT, paired acute and convalescent ASOT was collected in 18 non-purulent cases, but no purulent cases. A positive ASOT as defined above was noted in 84 of the 210 patients (40.0 %). The ASOT-positive rate was higher in the non-purulent than the purulent group (42.8 % vs. 11.1 %, *p* = 0.03).

**Table 3:** Diagnostic tests^a^ and associated results

	All (*n* = 465)	Non-purulent group (*n* = 369)	Purulent group (*n* = 96)	*P*-value
Diagnostic test^a^ performed (%)	432 (92.9)	338 (91.6)	94 (97.9)	0.032
Wound culture (%)	155 (33.3)	67 (18.2)	88 (91.7)	<0.001
Positive prevalence (%)	110 (71)	32 (47.8)	78 (88.6)	<0.001
Blood culture (%)	351 (75.5)	291 (78.9)	60 (62.5)	0.001
Positive prevalence (%)	33 (9.4)	24 (8.2)	9 (15)	0.103
ASOT (%)	210 (45.2)	192 (52.0)	18 (18.8)	<0.001
Positive prevalence (%)	84 (40.0)	82 (42.8)	2 (11.1)	0.031
One set	72 (34.3)	70 (36.5)	2 (11.1)	
Paired	12 (5.7)	12 (6.3)	0 (0.0)	
Puncture biopsy (%)	1 (0.2)	1 (0.3)	0 (0.0)	1.000
Positive prevalence (%)	0	0	0	

Data are number (%) of patients, unless otherwise indicated

*ASOT* antistreptolysin O titer

^a^It means blood culture, wound culture, ASOT, and puncture biopsy

### Microbiological findings

The etiological agent(s) were identified from one or more sites or by serology in 211 of the 465 patients (45.4 %) (Table 4). The causative bacteria were identified in 35.5 % of the non-purulent cases and 83.3 % of the purulent group. β-hemolytic streptococci were the predominant bacterial pathogens among patients with non-purulent cellulitis with positive microbiological findings, accounting for 70.2 % of cases (92/131). β-hemolytic streptococci were identified by wound culture (three cases, 3.3 %), ASOT (76 cases, 82.6 %), blood culture (seven cases, 7.6 %), or ASOT and blood culture (six cases, 6.5 %). The next most frequently identified bacteria in the non-purulent group were *S. aureus* (12.2 %), Gram-negative bacilli (9.9 %), and polymicrobial pathogens (4.6 %). *S. aureus* was the most common pathogen (60.0 %) amongst the 80 patients in the purulent group with positive microbiological findings, followed by polymicrobial pathogens (18.8 %), Gram-negative bacilli (12.5 %), and β-hemolytic streptococci (5.0 %). MRSA were more frequently isolated from the purulent than the non-purulent group (30.0 % vs. 6.1 %, *p* < 0.001). CA-MRSA, defined by both epidemiological criteria [17–19] reported the same number of cases, and were isolated in 21.3 % of cases in the purulent group and 3.8 % of cases in the non-purulent group (*p* = 0.681).

**Table 4:** Microbiological diagnosis in non-purulent (*n* = 131) and purulent cellulitis (*n* = 80) cases

	All (*n* = 211)	Non-purulent group (*n* = 131)	Purulent group (*n* = 80)	*P*-value
β-Hemolytic streptococci (%)	96 (45.5)	92 (70.2)	4 (5.0)	<0.001
*Staphylococcus aureus* ^a^ (%)	64 (30.3)	16 (12.2)	48 (60.0)	<0.001
MRSA^b^ (%)	32 (15.2)	8 (6.1)	24 (30.0)	<0.001
CA-MRSA^c^ (%)	22 (10.4)	5 (3.8)	17 (21.3)	0.681
GNB (%)	23 (10.9)	13 (9.9)	10 (12.5)	0.441
Polymicrobial pathogens (%)	21 (10.0)	6 (4.6)	15 (18.8)	0.001

Data are number (%) of patients, unless otherwise indicated

*CA-MRSA* community-associated methicillin-resistant *Staphylococcus aureus*, *GNB* Gram-negative bacilli

^a^Including methicillin-susceptible *S. aureus* and methicillin-resistant *S. aureus*

^b^Including CA-MRSA and non-CA-MRSA

^c^Defined by epidemiological criteria [17–19], not by genotypic criteria

### Antimicrobial susceptibility of MRSA stratified by CA-MRSA criteria

The antimicrobial susceptibility profiles of the 32 MRSA isolates were shown in Table 5. There was no significant difference in antimicrobial susceptibility between CA-MRSA and non-CA-MRSA isolates.

**Table 5:** Antibiotic susceptibilities of 32 MRSA isolates, stratified by CA-MRSA criteria

Antibiotic	No. (%) of samples, by susceptibility		
	Epidemiological classification		
	CA-MRSA (*n* = 22)	Non CA-MRSA (*n* = 10)	*P*-value
Chloramphenicol (%)	12 (54.5)	7 (70)	0.467
Clindamycin (%)^a^	4 (19)	0 (0)	0.287
Erythromycin (%)	4 (18.2)	0 (0)	0.283
Minocycline (%)	20 (95.2)	8 (80)	0.237
Levofloxacin (%)	18 (81.8)	5 (50)	0.096
Rifampin (%)	22 (100)	8 (80)	0.091
TMP-SMX (%)	21 (95.5)	7 (70)	0.079
Vancomycin (%)	22 (100)	10 (100)	1.000

Data are number (%) of patients, unless otherwise indicated

*TMP-SMX* trimethoprim-sulfamethoxazole

^a^Susceptibility to clindamycin was determined for 21 CA-MRSA isolates and nine non-CA-MRSA isolates. The D-test was performed to detect inducible clindamycin resistance for isolates found to be resistant to erythromycin, but susceptible to clindamycin

### Multivariable analyses of predictive factors for MRSA infection in 211 cases with a final pathogen diagnosis

Following adjustment for age, sex, sepsis, lower limb involvement, history of tinea pedis, diabetes, and wound, purulence was a positive predictor of MRSA infection (odds ratio (OR), 5.188; 95 % confidence interval (CI), 1.995–13.493; *p* = 0.001) (Table 6).

**Table 6:** Multivariable analysis of risk factors for MRSA infection in the 211 adult patients with final pathogen diagnosis

Variables	OR	95 % CI	*p* value
Age	0.994	0.974–1.014	0.529
Sex (male)	1.050	0.435–2.534	0.914
Sepsis	0.873	0.353–2.160	0.769
Lower limb involvement	0.777	0.283–2.137	0.625
Tinea pedis	0.490	0.102–2.366	0.375
Diabetes mellitus	0.391	0.105–1.455	0.161
Purulent group	5.188	1.995–13.493	0.001
Wound	1.122	0.420–2.994	0.819

### Empirical prescription of antimicrobial agents

The empirical prescription of antimicrobial agents is detailed in Table 7. In this study, the five most commonly prescribed antimicrobial agents were penicillin (29.5 %), oxacillin (28.6 %), cefazolin (19.4 %), β-lactam/β-lactamase inhibitors (3.7 %), and penicillin plus clindamycin (3.4 %). The prescription of antimicrobial agents differed significantly between the purulent and non-purulent groups. Penicillin was the most frequently prescribed agent in the non-purulent group (35.2 %), whereas oxacillin was the most frequently prescribed agent in the purulent group (39.6 %). The appropriate antimicrobial agent was more frequently prescribed in the non-purulent group than in the purulent group (83.2 % vs. 53.8 % of cases, *p* < 0.001). Among the 32 cases of MRSA identified, 12.5 % (4/32) of cases received the appropriate antibiotic therapy, and the prevalence of appropriate antimicrobial agent use was not significantly different between the purulent and non-purulent groups (16.7 % vs. 0 %, *p* = 0.55).

**Table 7:** Empirical prescription of antimicrobial agents. A. All cases of cellulitis B. Cases with pathogen isolated

A. Prescription of antimicrobial agents in all 465 cases of cellulitis				
	All (*n* = 465)	Non-purulent group (*n* = 369)	Purulent group (*n* = 96)	*P*-value
Penicillin (%)	137 (29.5)	130 (35.2)	7 (7.3)	<0.001
Oxacillin (%)	133 (28.6)	95 (25.7)	38 (39.6)	0.008
Cefazolin (%)	90 (19.4)	73 (19.8)	17 (17.7)	0.647
β-lactam/β-lactamase inhibitor (%)	17 (3.7)	9 (2.4)	8 (8.3)	0.012
Penicillin plus clindamycin (%)	16 (3.4)	16 (4.3)	0 (0.0)	0.052
Other (%)	72 (15.4)	46 (12.5)	26 (27.1)	<0.001
B. Prevalence of appropriate antimicrobial agent in 211 cases with pathogen isolated				
	All (*n* = 211)	Non-purulent group (*n* = 131)	Purulent group (*n* = 80)	*P*-value
Appropriate antimicrobial agent (%)	152 (72.0)	109 (83.2)	43 (53.8)	<0.001

Data are number (%) of patients, unless otherwise indicated

### Outcomes

The length of hospital stay and duration of antimicrobial therapy were shorter for the non-purulent group compared with the purulent group (8 vs. 11 days, *p* = 0.001; 13 vs. 17 days, *p* = 0.003, respectively). More patients underwent surgery in the purulent group than in the non-purulent group (49 % vs. 4.1 %, *p* < 0.001), and recurrence within 6 months post-infection was higher in the non-purulent group than in the purulent group (14.6 % vs. 3.1 %, *p* = 0.001). Three of the 465 patients (0.6 %) died within 6 months of discharge. None of the deaths were attributable to cellulitis.

## Discussion

The risk factors and the clinical and microbiological features of purulent and non-purulent cellulitis among hospitalized Taiwanese adults in the current study were remarkably similar to those reported in Western countries [20–24]. In the present study, patients with non-purulent cellulitis were more likely to have lower limb involvement, stasis dermatitis, and tinea pedis, along with a higher likelihood of recurrence than those with purulent cellulitis. Patients with purulent cellulitis were more likely to have wounds than those with non-purulent cellulitis. As in other studies, β-hemolytic streptococci were the predominant pathogens in the non-purulent group [2, 25], and *S. aureus* was the most common pathogen in the purulent group [11].

Cellulitis is a relatively common infectious disease in both children and adults. To ensure proper treatment, it needs to be differentiated from focal infections with surrounding inflammation, such as septic bursitis, osteomyelitis and septic arthritis, and non-infectious inflammatory diseases. We excluded 503 of the original 968 patients (52.0 %) because of misdiagnoses. The three most common misdiagnosed infections were anorectal abscess (*n* = 84), deep layer involvement (*n* = 64), and odontogenic infection (*n* = 37). The three most common misdiagnosed non-infectious diseases were phlebitis (*n* = 42), deep vein thrombosis (*n* = 7), and tumor with central necrosis (*n* = 5).

A large study conducted in emergency departments across the United States demonstrated that 76 % of cases of purulent soft tissue infection were caused by *S. aureus*, 59 % of which were CA-MRSA [11]. The present study demonstrates similar results. Of the purulent cases in which the etiological agent was identified, 60 % were caused by *S. aureus*, 35.4 % of which were CA-MRSA. A prospective study of 179 patients with non-purulent cellulitis based on acute and convalescent sera for ASO and DNase-B antibodies found that 73 % of cases had serological evidence of β-hemolytic *Streptococcal* infection [2]. A recently published prospective study of 77 cases with non-necrotizing cellulitis using serological study of *Streptococcus* also demonstrated similar results [25]. In the present study, however, the serological evidence of streptococcus infection may be underestimated in the non-purulent group because paired acute and convalescent ASOT were collected in only 18 cases, 66.7 % (12/18) of which were positive. 90.6 % (174/192) cases with measured ASOT in the non-purulent group received only a single ASOT, and the prevalence of positive ASOT was 40.2 % (70/174).

An increased ASOT between the acute and convalescent phases provides a more accurate reflection of a preceding streptococcal infection than a single titer [26]. However, it is not always feasible to obtain paired sera. Therefore, the occurrence of a single isolated titer that is higher than the upper limit of normal value is also evidence of a previous streptococcal infection. In patients with group A streptococcal infections, ASOT begins to rise after approximately 1 week, reaching maximal levels at 3–6 weeks post-infection [27, 28], and begins to decline in uncomplicated infections at 6–8 weeks post-infection, although in some patients the titer may remain elevated for indefinite periods [29]. We reviewed the charts of the 70 non-purulent cases with a single positive ASOT in the current study, which showed that only six of these cases experienced another episode of cellulitis in the year preceding the current episode. These findings provide further support for β-hemolytic streptococci being the causal organism in the majority of the 70 cases with a single positive ASOT.

The lower rate of ASOT performed in the purulent group compared with the non-purulent group may have led to an underestimation of the incidence of *Streptococcus* infection (purulent vs. non-purulent, 18.8 % vs. 52.0 %, *p* <0.001). This selection bias is unavoidable in a retrospective study, but may be limited. First, the lower rate of ASOT testing in the purulent group may reflect the fact that these patients did not present with typical *Streptococcus* cellulitis, which is characterized by rapidly spreading areas of inflammation in the lower extremities, sometimes accompanied by lymphangitis and regional lymphadenitis in cases with predisposing conditions, such as tinea pedis, stasis dermatitis, compromised lymphatic drainage, and venous insufficiency. Second, the prevalence of positive ASOT, whether as single or paired results, was significantly lower in the purulent group than in the non-purulent group (purulent vs. non-purulent, 11.1 % vs. 42.75 %, *p* = 0.031).

In the present study, cellulitis cases were treated by a range of practitioners, including primary care, emergency, and other specialist physicians. Multiple antibiotics were empirically prescribed for cellulitis, and these differed significantly between the non-purulent and purulent groups (Table 7). The rate of prescription of inappropriate antimicrobial agents was higher in the purulent group than in the non-purulent group (46.2 % vs. 16.8 %, *p* < 0.001). Overuse of antibiotics is associated with increased antibiotic resistance and cost of treatment [30, 31]. However, inappropriately withholding necessary antibiotics may have adverse effects on outcomes. The impact of inappropriate antimicrobial agent use on length of hospital stay, duration of antimicrobial therapy and medical cost needs further analysis.

There has been much discussion as to whether coverage for CA-MRSA should be included in empirical regimens for cellulitis. Published guidelines offer different recommendations. The 2011 IDSA guidelines for MRSA management recommend antimicrobial therapy targeting β-hemolytic streptococci for non-purulent cellulitis, and specific CA-MRSA therapy for cases of purulent cellulitis [15]. The 2014 IDSA guidelines for skin and soft tissue infection management recommend antimicrobial therapy effective against both MRSA and streptococci for patients whose cellulitis is associated with penetrating trauma, evidence of MRSA infection elsewhere, nasal colonization with MRSA, injection drug use, or systemic inflammatory response syndrome [16]. However, there are several issues that need to be addressed to provide an optimal approach for the use of antibiotics in the management of cellulitis. The diagnosis and management of cellulitis depend largely on clinical features. Clinicians are often unwilling to pursue microbiological diagnosis because of the absence of culturable material in most cases of non-purulent cellulitis, the relatively low recovery rate of pathogens [1], and favorable prognosis in most cases following empirical therapy. Some primary physicians may use a strategy known as double coverage, which refers to the use of at least two antibiotics with Gram-positive coverage (such as trimethoprim-sulfamethoxazole plus cephalexin) to target MRSA, methicillin-sensitive *S. aureus*, and β-hemolytic streptococci [32]. However, a recent randomized, multicenter, double-blinded, placebo-controlled trial demonstrated no improvement in outcomes among patients with uncomplicated (non-purulent) cellulitis treated with trimethoprim-sulfamethoxazole in addition to cephalexin compared with those treated with cephalexin alone [33].

In the current study, MRSA was more frequently isolated from the purulent group than the non-purulent group (MRSA: 30.0 % vs. 6.1 %, *p* < 0.001). In multivariable analyses of factors that could be used for prediction of MRSA infection, only purulence was a positive predictor of MRSA (OR, 5.188; 95 % CI, 1.995–13.493; *p* = 0.001). The lack of a significant difference in prevalence of CA-MRSA between the two groups may be caused by the small number of isolates (CA-MRSA: purulent vs. non-purulent: 21.3 % vs. 3.8 %, *p* = 0.681). Initial empirical antimicrobial therapy, when prescribed, must be chosen based on local antimicrobial sensitivity data. ST59-MRSA-VT/IV is the predominant CA-MRSA clone in Taiwan [34, 35]. This clone is typically resistant to erythromycin, clindamycin, and occasionally, gentamicin, but is susceptible to trimethoprim-sulfamethoxazole, tetracyclines, and fluoroquinolones [34]. Because of the retrospective nature of the present study, we were unable to access the bacterial isolates for molecular epidemiological studies. However, the antimicrobial sensitivity testing of the 22 CA-MRSA isolates provided similar results.

The current study provides strong support for the clinical approach recommended in the 2011 IDSA guidelines, dividing cellulitis into purulent and non-purulent groups to help select the appropriate empirical antimicrobial therapy to be applied in Taiwan. This consists of β-lactam agents directed at streptococci for non-purulent cellulitis, and antibiotics active against MRSA for purulent cellulitis.

The major limitation of this study is information bias, which is inherent in all retrospective studies. It also unavoidably contributes to selection bias because not all patients received paired ASOT testing, and none of the patients underwent a serological test for *S. aureus*. Both of these factors may lead to an underestimate of the role of β-hemolytic streptococci and *S. aureus* as etiological agents of cellulitis. Additionally, our inability to differentiate cutaneous abscess from purulent cellulitis may have overestimated the number of cases in the purulent group. The current study also only examined patients from one hospital over a 1-year period. A multicenter-study is needed to generalize our findings to all of Taiwan. Prospective studies are needed to assess changes that might occur regarding the predominant microorganisms, and the emergence of antibiotic-resistant bacteria. Finally, the patients in this study were limited to hospitalized cases, meaning that infections were more severe. Therefore, the results of this study cannot be generalized to all patients with cellulitis in Taiwan.

## Conclusions

In conclusion, the epidemiology, clinical features, and microbiology of purulent and non-purulent cellulitis were significantly different in hospitalized Taiwanese adults. Understanding the local epidemiology and etiological agents is essential for the development of guidelines for empirical antimicrobial therapy of cellulitis in Taiwan and other countries in South East Asia.

## Abbreviations

ASOT: Antistreptolysin O titer
CA-MRSA: Community-associated methicillin-resistant *Staphylococcus aureus*
CK: Creatine kinase
Cr: Creatinine
CRP: C-reactive protein
GOT: Glutamate oxaloacetate transaminase
HA-MRSA: Healthcare-associated methicillin-resistant *Staphylococcus aureus*
Hb: Hemoglobin
ICD-9-CM: International Classification of Disease, Ninth Revision, Clinical Modification
IDSA: Infectious Diseases Society of America
PLT: Platelet count
SCCmec: Staphylococcal cassette chromosome *mec*
SD: Standard deviation
